# Mutation load estimation model as a predictor of the response to cancer immunotherapy

**DOI:** 10.1038/s41525-018-0051-x

**Published:** 2018-04-30

**Authors:** Guan-Yi Lyu, Yu-Hsuan Yeh, Yi-Chen Yeh, Yu-Chao Wang

**Affiliations:** 1 Institute of Biomedical Informatics, National Yang-Ming University, Taipei, 11221 Taiwan; 2 Department of Life Sciences and Institute of Genome Sciences, National Yang-Ming University, Taipei, 11221 Taiwan; 3 Department of Pathology and Laboratory Medicine, Taipei Veterans General Hospital, Taipei, 11217 Taiwan; 4 School of Medicine, National Yang-Ming University, Taipei, 11221 Taiwan; 5 Center for Systems and Synthetic Biology, National Yang-Ming University, Taipei, 11221 Taiwan

## Abstract

The determination of the mutation load, a total number of nonsynonymous point mutations, by whole-exome sequencing was shown to be useful in predicting the treatment responses to cancer immunotherapy. However, this technique is expensive and time-consuming, which hampers its application in clinical practice. Therefore, the objective of this study was to construct a mutation load estimation model for lung adenocarcinoma, using a small set of genes, as a predictor of the immunotherapy treatment response. Using the somatic mutation data downloaded from The Cancer Genome Atlas (TCGA) database, a computational framework was developed. The estimation model consisted of only 24 genes, used to estimate the mutation load in the independent validation cohort precisely (*R*^2^ = 0.7626). Additionally, the estimated mutation load can be used to identify the patients with durable clinical benefits, with 85% sensitivity, 93% specificity, and 89% accuracy, indicating that the model can serve as a predictive biomarker for cancer immunotherapy treatment response. Furthermore, our analyses demonstrated the necessity of the cancer-specific models by the constructed melanoma and colorectal models. Since most genes in the lung adenocarcinoma model are not currently included in the sequencing panels, a customized targeted sequencing panel can be designed with the selected model genes to assess the mutation load, instead of whole-exome sequencing or the currently used panel-based methods. Consequently, the cost and time required for the assessment of mutation load may be considerably decreased, which indicates that the presented model is a more cost-effective approach to cancer immunotherapy response prediction in clinical practice.

## Cancer genetics: Predicting patient response to immunotherapy

Estimating patients’ mutation load from a small set of genes can accurately predict their response to cancer immunotherapy. Harnessing patients’ immune response to target tumor cells is an effective treatment approach in some cases but not others. A patient’s number of deleterious genetic mutations across all their protein-coding genes has been shown to correlate with their responsiveness to immunotherapy. However, whole-exome sequencing is time-consuming and costly. Yu-Chao Wang *at the* National Yang-Ming University, Taiwan, and colleagues have developed cancer-specific mutation load estimation models for adenocarcinoma, melanoma and colorectal cancer that require sequencing only a small number of genes. They show that the mutation load in lung adenocarcinoma patients can be estimated from 24 genes and that they can predict immunotherapy responsiveness with similar accuracy to that obtained using whole-exome sequencing.

## Introduction

Cancer is the leading cause of human deaths worldwide. Cancer therapeutics are intensively studied, and immunotherapy represents one of the novel promising therapeutic approaches. In this type of therapy, the immune system is recruited to fight against tumor development and expansion, and the most successful immunotherapeutics to date have been immune checkpoint inhibitors, such as anti-programmed cell death protein 1 (PD-1), anti-PD-L1, and anti-CTLA-4 antibodies.^1^ Under normal conditions, T-cells can identify and kill tumor cells by recognizing the antigens on tumor cells. However, one tumor cell mechanism, which allows them to avoid killing by taking advantage of the tightly regulated nature of T-cells, has evolved. Specifically, PD-1, the surface receptor on T-cells, is an immune checkpoint molecule responsible for avoiding autoimmunity. Upon the binding of PD-1 to its ligand, PD-L1, the T-cells are deactivated. Therefore, tumor cells can present PD-L1 on their surfaces and escape death by deactivating T-cells.^2^ Immune checkpoint inhibitors have been developed to block the interaction between PD-1 and PD-L1, allowing the immune system to act against tumor.^3^ US Food and Drug Administration (FDA) have approved anti-PD-1 (nivolumab, pembrolizumab), anti-PD-L1 (atezolizumab), and anti-CTLA-4 (ipilimumab) drugs for the treatment of different kinds of cancers, such as melanoma, non-small-cell lung cancer, bladder cancer, head and neck cancer, and renal cell carcinoma.^4–6^ Clinical trials, examining the anti-tumor activity of PD-1/PD-L1 blocking antibodies against other solid and hematological malignancies are in progress, demonstrating that the PD-1 pathway represents a promising target for anti-cancer therapy.^7^

Although the efficacy of immunotherapy has been demonstrated, treatment response is only observed in a subset of patients.^8–10^ Therefore, the identification of patients that can potentially respond to drugs and the understanding of the underlying mechanisms are necessary. Rizvi et al.^10^ demonstrated that the mutation load, the number of nonsynonymous point mutations, may be a useful predictive biomarker for treatment response. An increased number of nonsynonymous point mutations is associated with improved objective response, durable clinical benefit (DCB), and progression-free survival (PFS). However, whole-exome sequencing, necessary for the determination of mutation load is not sufficiently cost and time-effective to be applied as a standard clinical test. In contrast, cancer gene panels composed of about 300–600 cancer-related genes are used in clinical practice to investigate the genetic profile of tumors.^11,12^ Therefore, the application of the next-generation sequencing (NGS) gene panels for the precise estimation of the mutation load and treatment response prediction was investigated. Johnson et al.^13^ showed that the mutation counts detected in the 315-gene NGS panel for melanoma are highly correlated with those assessed by whole-exome sequencing (Spearman correlation coefficient = 0.995). Additionally, patients with high mutation counts detected by NGS gene panels were demonstrated to have a significantly higher PFS than those with the low gene panel mutation counts.^12^ Further, Roszik et al.^14^ developed a novel algorithmic method to accurately predict total mutation load within tumors using approximately 170 genes in the NGS panels. These results indicate that the NGS gene panels with hundreds of genes can be used to estimate the mutation load of tumors and to predict the efficacy of immunotherapy. However, Campesato et al.^12^ further demonstrated that the predictive accuracy is apparently lost when the number of genes in the NGS panel is lower than 150, suggesting that the comprehensive gene panels, comprising more than 300 cancer-related genes, should be employed. Unfortunately, the cost of the NGS gene panels with more than 300 genes is still high, and this may be unattainable for the routine clinical tests in most hospitals worldwide.

Here, based on the publicly available cancer genomics information, we proposed a computational framework for the construction of a mutation load estimation model for lung adenocarcinoma, the most common type of lung cancer, and we analyzed the effectiveness of this model for the prediction of cancer immunotherapy response. Furthermore, the computational framework was applied to construct the mutation load estimation models for melanoma and colorectal cancer, respectively. These cancer-specific models may allow the design of customized panels for the targeted sequencing of selected genes to estimate mutation load, instead of whole-exome sequencing, decreasing the cost and time required for the assessment of mutation load.

## Results

### Computational framework overview

**Fig. 1:**
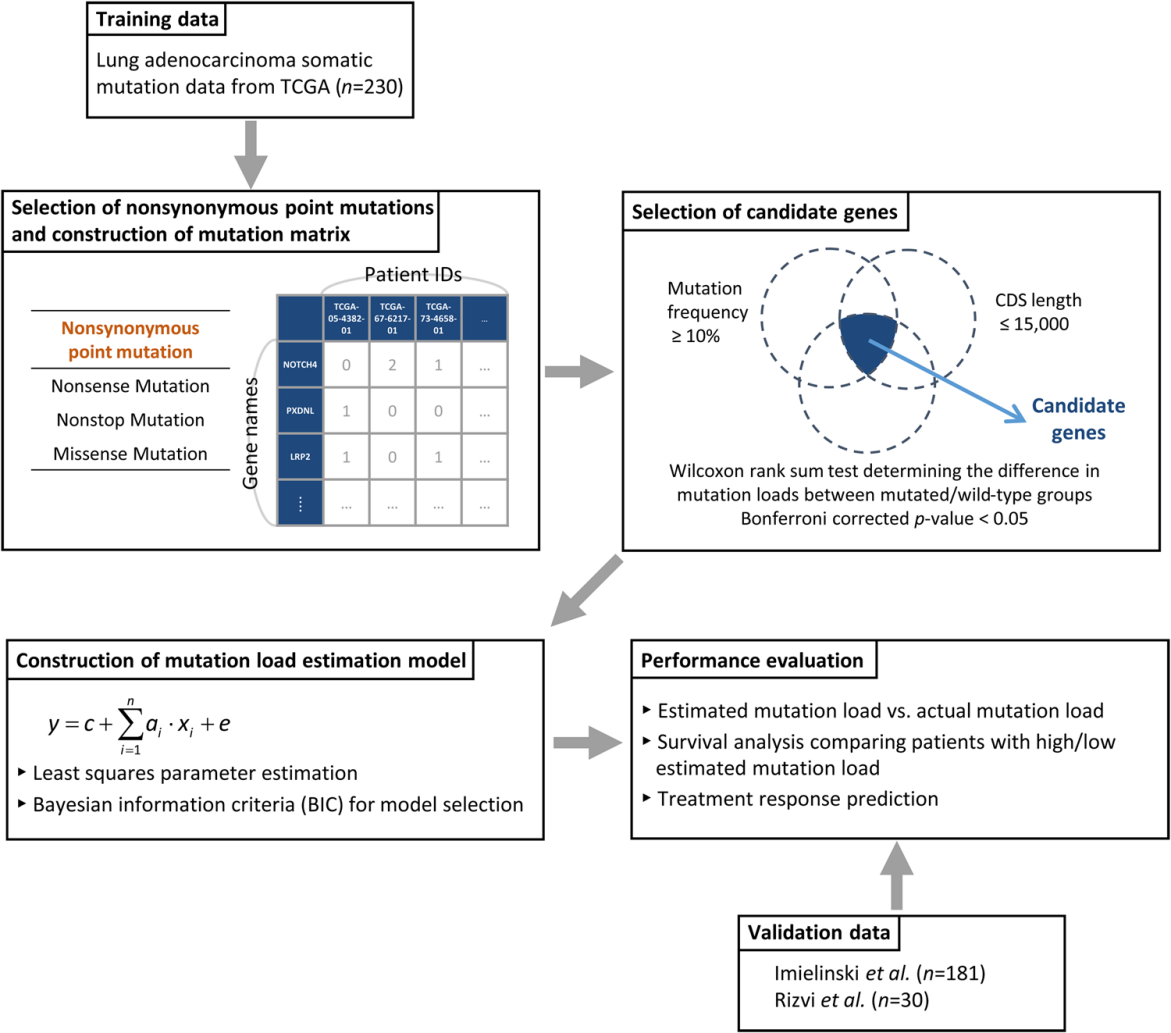
Computational framework used during the construction of the lung adenocarcinoma mutation load estimation model

The flowchart of the computational framework used during the mutation load estimation model construction for lung adenocarcinoma is shown in Fig. 1. We generated the mutation matrix with the somatic mutation data downloaded from The Cancer Genome Atlas (TCGA)^15^ as the training data. Subsequently, the candidate genes were selected based on a set of defined criteria. Afterward, a simple linear model was used for the construction of mutation load estimation model. Least squares parameter estimation method was employed for parameter identification and Bayesian information criterion (BIC) was used for model selection. After the selection of the most appropriate model, the performance of the mutation load estimation model was evaluated and verified using the mutation information obtained from the independent validation data. Details of this procedure are presented in Materials and methods.

### Mutation load estimation model for lung adenocarcinoma was constructed using only 24 genes

With the lung adenocarcinoma somatic mutation data downloaded from TCGA database, a computational framework was developed to construct the mutation load estimation model. After selecting nonsynonymous point mutations, the mutation matrix with 13,526 genes and 230 patients was generated. Subsequently, based on the defined selection criteria (mutation frequency ≥ 10%, coding DNA sequence (CDS) length ≤ 15,000, and Bonferroni corrected *p*-value < 0.05 in Wilcoxon test), 62 candidate genes were selected (Materials and methods, Supplementary Fig. 1, and Supplementary Table 1).

For the 62 candidate genes selected, there are 2^62^−1 combinations of gene sets, resulting in 2^62^−1 possible models. Based on the least squares parameter estimation and BIC for model selection (Materials and methods, Supplementary Methods), the most appropriate mutation load estimation model for lung adenocarcinoma was shown to contain only 24 genes, selected as follows:

1 $$\begin{array}{l} \hat y{{ = }}68.72 \cdot {\mathrm{{\it PXDNL} + }}64.27 \cdot {\mathrm{{\it NOTCH4} + }} \cdots {{ + }}27.1 \cdot {\mathrm{\it PAPPA2}} \\ + 22.57 \cdot {\mathrm{{\it ZFHX4} + }}47.24, \end{array}$$

where $\hat y$ is the estimated mutation load using the 24-gene model. The complete list of genes and their corresponding parameters in the constructed estimation model are shown in Table 1. With the model constructed as shown by equation (1), the mutation counts in these 24 genes of a patient allow the estimation of the mutation load.

**Table 1:** Genes and the corresponding parameters used in the constructed lung adenocarcinoma mutation load estimation model

Gene symbol	Entrez ID	Parameter	Gene symbol	Entrez ID	Parameter
*PXDNL*	137902	68.72	*ASXL3*	80816	39.52
*NOTCH4*	4855	64.27	*ERICH3*	127254	37.88
*CSMD2*	114784	58.51	*HRNR*	388697	37.14
*PLPPR4*	9890	54.54	*LRP2*	4036	36.12
*NRXN1*	9378	50.57	*ASTN1*	460	35.65
*KMT2C*	58508	49.19	*RYR3*	6263	35.57
*ADAMTS12*	81792	46.68	*MXRA5*	25878	34.54
*COL6A3*	1293	45.95	*ADGRG4*	139378	31.22
*ZNF831*	128611	41.88	*NALCN*	259232	29.79
*FAM135B*	51059	41.01	*LRP1B*	53353	28.99
*FLG*	2312	40.99	*PAPPA2*	60676	27.10
*FAM47C*	442444	40.62	*ZFHX4*	79776	22.57
			Constant term		47.24

### The constructed model for lung adenocarcinoma can be used for the precise estimation of the mutation load and accurate prediction of the immunotherapy treatment response

**Fig. 2:**
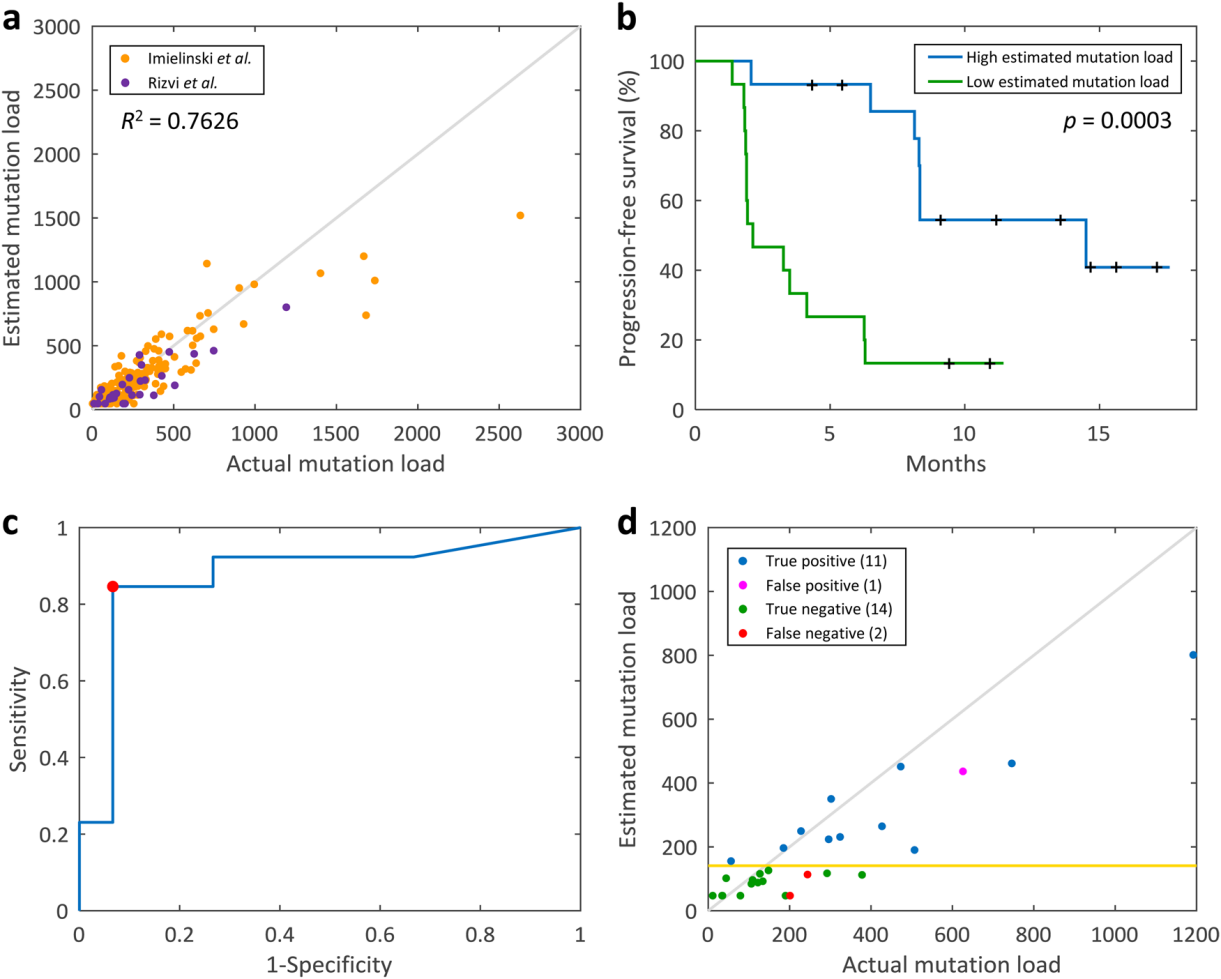
Performance evaluation of the mutation load estimation model. **a** Estimated mutation load vs. actual mutation load using the independent validation data (*n* *=* 211). **b** Survival analysis comparing PFS in patients with the high estimated mutation loads (*n* = 15) with those with the low estimated mutation loads (*n* = 15). The log-rank test results indicate that the higher estimated mutation load correlates with improved PFS (*p* = 0.0003). **c** ROC curve for the classification of DCB/NDB patients using the estimated mutation load. The red point indicates the optimal discrimination threshold 141. AUC = 0.8744. **d** Immunotherapy response prediction using the estimated mutation load. Gold horizontal line represents the optimal discrimination threshold, 141

For the performance evaluation of the constructed model for lung adenocarcinoma, the mutation load for all patients in the training data from TCGA (*n* *=* 230) was estimated using this model. *R*^*2*^ between the estimated and actual mutation load was shown to be 0.9336 (Supplementary Fig. 2), indicating that the estimated mutation loads highly correlate with the actual mutation loads. Additionally, in order to validate the constructed mutation load estimation model, two independent validation datasets (*n* *=* 211) were applied as well, to test the performance (Materials and methods),^10,16^ and *R*^2^ between the estimated and actual mutation load was shown to be 0.7626 for the independent validation cohort (Fig. 2a).

We analyzed the performance of the mutation load estimation model for lung adenocarcinoma, in the prediction of the immunotherapy treatment response, using information from an independent validation cohort.^10^ The actual and estimated mutation loads of the patient subgroups with different clinical characteristics are presented in Supplementary Figs. 3 and 4, respectively. Survival analysis was applied for the comparison of the PFS between the patients (*n* *=* 30) with high/low estimated mutation loads, and we demonstrated that a high mutation load, as estimated using our constructed model, was significantly associated with the improved PFS (*p* = 0.0003, log-rank test) (Fig. 2b). Univariate analysis showed that strong PD-L1 expression and high mutation load (either actual or estimated mutation load) are significantly associated with the improved PFS. In multivariate analysis, after adjusting for the PD-L1 expression, high estimated mutation load remained significantly associated with improved PFS (Supplementary Table 2). The estimated mutation loads were also employed to predict whether the patients have DCBs or no durable benefits (NDBs) following the immunotherapy. To this end, we determined a discrimination threshold first. Because the higher estimated mutation load correlates with the improved PFS, if the estimated mutation load of a patient is higher than or equal to the discrimination threshold, that patient is more likely to have DCB, and vice versa. Therefore, the receiver operating characteristic (ROC) curve was used to determine the optimal discrimination threshold, and the estimated mutation load ≥ 141 was identified as the threshold combining the maximal sensitivity and specificity. The area under the curve (AUC) for DCB/NDB classification using our constructed model was shown to be 0.8744, demonstrating that the estimated mutation load can predict the immunotherapy treatment response quite well (Fig. 2c). According to the estimated mutation load of each lung adenocarcinoma patient and the identified optimal threshold, the sensitivity and specificity of DCB/NDB classification using our constructed model were shown to be 0.8462 and 0.9333, respectively (Fig. 2d). Furthermore, the accuracy of the cancer immunotherapy response prediction was 0.8929, obtained using our estimation model, which is comparable to that obtained using whole-exome sequencing.^10^

### Performance verification by random models

**Fig. 3:**
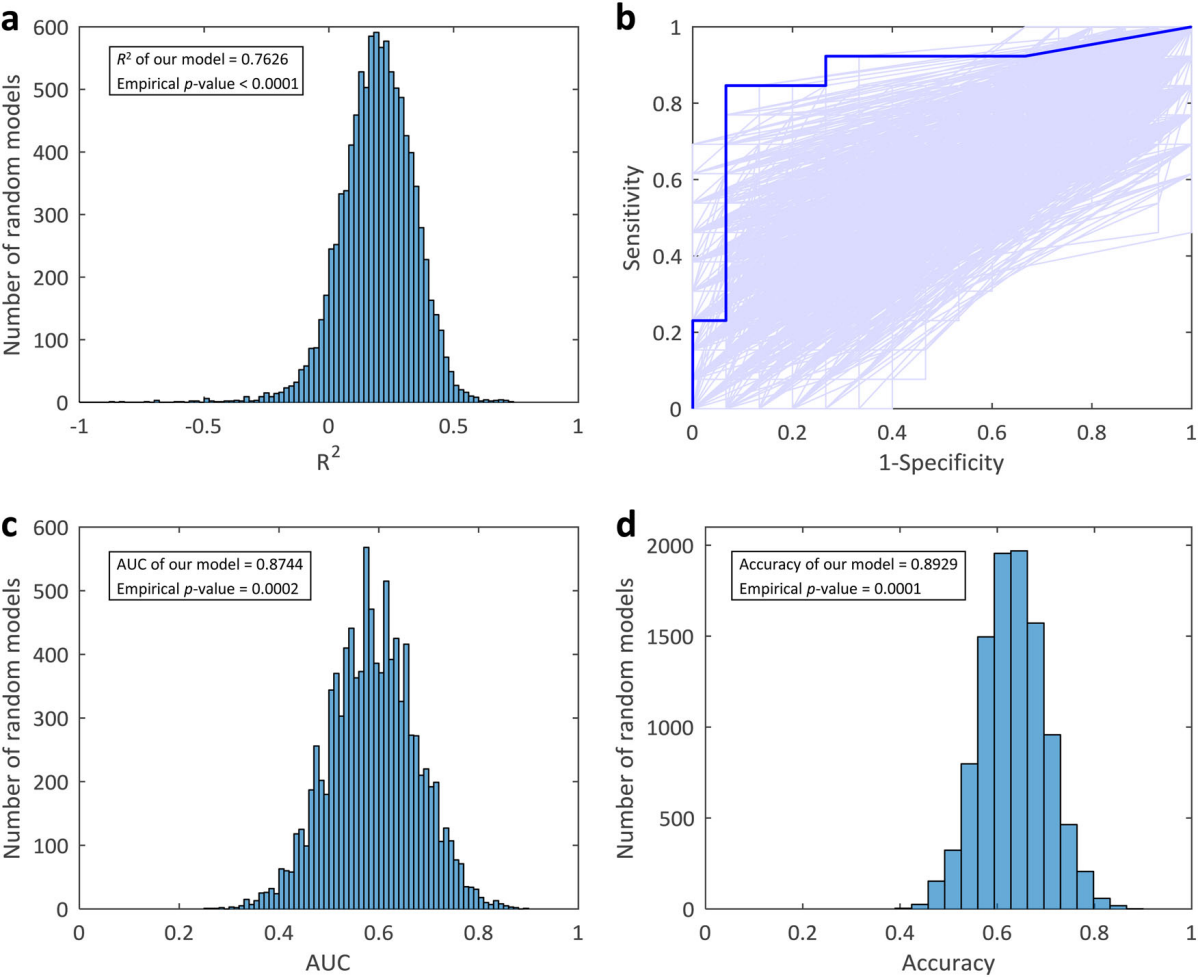
Performance verification using 10,000 random models. **a** Empirical distribution of *R*^2^ between the estimated and actual mutation load for 10,000 random models. **b** ROC curves for the constructed model and 10,000 random models. Blue line, the ROC curve of classifier based on the mutation load estimation model. **c** Empirical distribution of AUC statistic for 10,000 random models. **d** Empirical distribution of the classification accuracy for 10,000 random models

Although we demonstrated that our estimation model for lung adenocarcinoma can be used for the precise estimation of the mutation load of a patient, and the estimated mutation load is useful for the prediction of cancer immunotherapy treatment response, we further verified the results, comparing them with the results of a model constructed using 24 randomly selected genes. Therefore, 24 genes were randomly selected from the generated mutation matrix, and a random model was constructed with the help of the least squares parameter estimation method. The procedure was repeated 10,000 times, resulting in 10,000 random models. Subsequently, the performances of these random models were evaluated. The empirical distribution of *R*^2^ between the estimated and actual mutation loads in the independent validation cohort for 10,000 random models is presented in Fig. 3a. The *R*^2^ of our constructed model (0.7626) was shown to be higher than all *R*^2^ calculated by random models, and the empirical *p*-value of *R*^2^ was *p* < 0.0001. Further, based on the random models and the immunotherapy treatment response data, the ROC curves for all 10,000 random models were plotted (Fig. 3b) and the empirical distribution of AUC is shown in Fig. 3c (empirical *p-*value = 0.0002). For each random model, the optimal discrimination threshold can also be identified using the ROC curve, allowing the determination of the classification accuracy. The empirical distribution of classification accuracy for 10,000 random models is displayed in Fig. 3d and the empirical *p-*value of our constructed model was 0.0001.

### Cancer-type-specific mutation load estimation model is necessary for clinical application

**Fig. 4:**
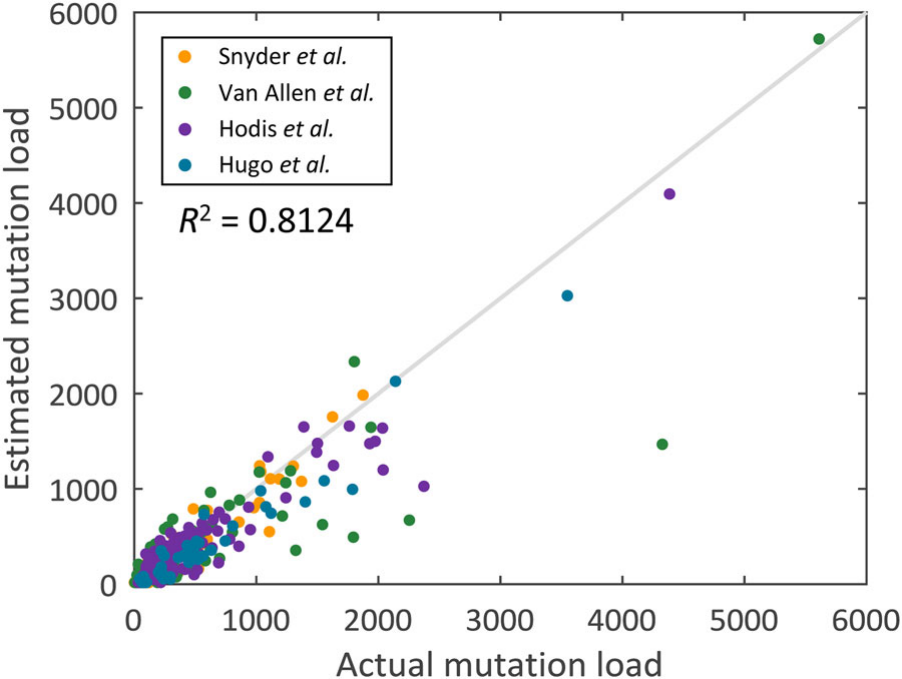
Performance evaluation comparing the actual mutation load with the estimated mutation load using the melanoma model in an independent validation cohort (*n* *=* 333)

**Fig. 5:**
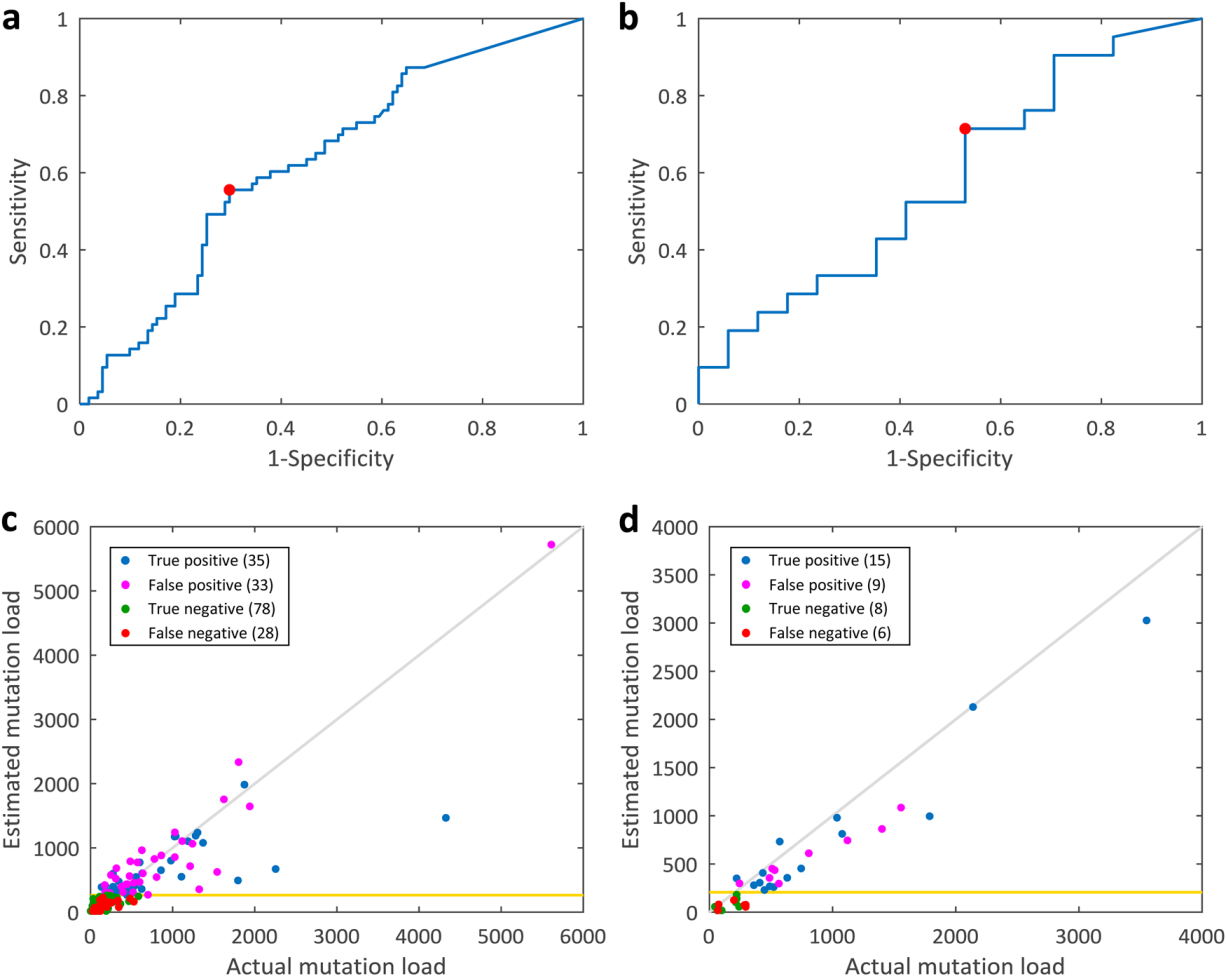
Performance evaluation of the immunotherapy treatment response prediction using the melanoma mutation load estimation model. **a** ROC curve for the classification of clinical benefits using the estimated mutation load in the anti-CTLA-4 treatment patients. Red point, the optimal discrimination threshold 264. AUC = 0.6270. **b** ROC curve for the anti-PD-1 treatment patients. Red point, the optimal discrimination threshold 206. AUC = 0.5812. **c** Immunotherapy response prediction using the estimated mutation load for the anti-CTLA-4 treatment patients. Gold horizontal line represents the optimal discrimination threshold 264. The accuracy of the classification is 0.6494. **d** Immunotherapy response prediction for the anti-PD-1 treatment patients. Gold horizontal line represents the optimal discrimination threshold 206. The accuracy of the classification is 0.6053

In addition to lung adenocarcinoma, previous studies showed that the tumor mutation load is associated with the degree of clinical benefit of immunotherapy in melanomas.^17,18^ Therefore, we investigated whether the mutation load estimation model developed using lung adenocarcinoma data can also be employed to estimate the mutation load and predict immunotherapy treatment response for melanoma patients. Mutation data of the melanoma patients^17–20^ and the clinical outcomes for patients treated with anti-CTLA-4^17,18^ or anti-PD-1^20^ agents were retrieved. The constructed 24-gene estimation model of lung adenocarcinoma was applied to estimate the mutation load of these melanoma patients. *R*^2^ between the estimated mutation load and actual mutation load was shown to be 0.6574, and the accuracy of classification with the optimal discrimination threshold was shown to be 0.6437 and 0.6579 for anti-CTLA-4 and anti-PD-1 treatments, respectively. These results demonstrate that the mutation load estimation model trained for lung adenocarcinoma can be used for the estimation of the mutation load and the immunotherapy treatment response prediction in melanoma patients to a certain extent. However, the performance of the constructed model was not as good as that for lung adenocarcinoma patients. To test whether a melanoma mutation estimation model can yield better results, we utilized the somatic mutation data of melanoma patients obtained from TCGA database (*n* = 333)^21^ to train a melanoma mutation load estimation model, using the same approaches as the one used for the lung adenocarcinoma patients. The constructed melanoma model contained 22 genes (Table 2). *R*^2^ between the estimated mutation load and actual mutation load in an independent validation cohort collected from four studies (*n* = 333)^17–20^ was shown to be 0.8124 (Fig. 4), which is superior to that calculated using the lung adenocarcinoma model, indicating that cancer-type-specific mutation load estimation models are necessary. Additionally, clinical responses in the melanoma patients treated with anti-CTLA-4^17,18^ or anti-PD-1^20^ agents were acquired to assess the performance of the immunotherapy response prediction. The actual and estimated mutation loads of patient subgroups with different clinical characteristics are presented in Supplementary Figs. 5 and 6, respectively. Overall survival (OS) for both anti-CTLA-4 and anti-PD-1 treatments were shown to have no significant correlation with the estimated mutation load (Supplementary Fig. 7). For the anti-CTLA-4 treatment patients, AUC for the classification of clinical benefit using estimated mutation load was 0.6270 (Fig. 5a), and the accuracy of classification with the optimal discrimination threshold was shown to be 0.6494 (Fig. 5c). Univariate analysis showed that M category, serum lactate dehydrogenase (LDH) level, prior courses of systemic therapy, and mutation load (either actual or estimated mutation load) are significantly associated with clinical benefit. In multivariate analysis, after adjusting for M category, LDH level and prior systemic therapy, estimated mutation load was shown to remain significantly associated with the clinical benefit (Supplementary Table 3). Furthermore, AUC for the classification of clinical benefits using estimated mutation load for the anti-PD-1 treatment patients was 0.5812 (Fig. 5b), and the accuracy of classification was shown to be 0.6053 (Fig. 5d). However, there were no significant differences in the mutation load between the treatment responders and non-responders (Supplementary Table 4). These results indicate that the melanoma model can estimate the mutation load of the melanoma patients more precisely than the lung adenocarcinoma model. Unexpectedly, however, the prediction accuracy of the treatment response of melanoma model was similar to that of the lung adenocarcinoma model. In addition to the lung adenocarcinoma and melanoma, the proposed computational framework was also applied for colorectal cancer, where mutation load estimation is currently not available. Somatic mutation information downloaded from TCGA was used as the training data (*n* = 536)^22^ and the constructed colorectal mutation load estimation model contained 22 genes (Supplementary Table 5). The mutation data for colorectal cancer patients from two independent studies (*n* = 691)^23,24^ were employed as the validation data. *R*^2^ between the estimated mutation load and actual mutation load was shown to be 0.8794 (Supplementary Fig. 8). The actual and estimated mutation loads of patient subgroups with different clinical characteristics are presented in Supplementary Figs. 9 and 10, respectively. Since no immunotherapy response data for these colorectal cancer samples exist, the treatment response prediction accuracy of the constructed colorectal model cannot be evaluated.

**Table 2:** Genes and the corresponding parameters used in the constructed melanoma mutation load estimation model

Gene symbol	Entrez ID	Parameter	Gene symbol	Entrez ID	Parameter
*TNXB*	7148	93.24	*RYR2*	6262	50.95
*NPAP1*	23742	80.88	*LRP2*	4036	49.34
*DNAH10*	196385	75.59	*COL4A4*	1286	41.92
*ADGRG4*	139378	69.31	*RP1*	6101	40.24
*SCN10A*	6336	58.65	*APOB*	338	38.24
*CMYA5*	202333	55.98	*UNC13C*	440279	36.52
*FAT3*	120114	54.53	*XIRP2*	129446	35.84
*ZNF831*	128611	53.47	*MXRA5*	25878	33.81
*CSMD3*	114788	53.14	*DNAH11*	8701	30.16
*MYH4*	4622	52.94	*MUC17*	140453	28.28
*PKHD1L1*	93035	51.74	*DNAH9*	1770	27.21
			Constant term		18.17

## Discussion

Immunotherapy using immune checkpoint inhibitors has emerged as a promising new therapeutic approach to cancer treatment in recent years. However, there are still patients who do not respond to this type of therapy, and the potential predictive biomarkers that can be used to identify the potential responders of immunotherapy are intensively studied, since this information can support the medical decision-making. Previous studies demonstrated that the mutation load measured by whole-exome sequencing may predict the sensitivity to cancer immunotherapy. However, due to the high costs and technical threshold, the routine use of whole-exome sequencing is generally not feasible in medical institutions, which hinders the application of this method as a standard clinical test. In this study, we developed a computational framework for the construction of the mathematical model that can be used for the estimation of the patient mutation load using the genetic information on a small number of genes. The constructed mutation load estimation model for lung adenocarcinoma using only 24 genes was shown to allow the precise estimation of the mutation load and the highly accurate prediction of the immunotherapy response in the lung adenocarcinoma patients, and this accuracy was shown to be similar to that of the whole-exome sequencing. Furthermore, all performance indices demonstrated that our mutation load estimation model outperforms the random models, which shows the effectiveness of the computational framework proposed in this study.

Previous studies showed that the commercial or institutional gene panels that consist of genes known or suspected to be relevant to cancer can be used to estimate the mutation load.^25^ However, the number of genes in these panels is considerably higher than that in our model, including as many as 170, 315, and 641 genes.^12,14^ Additionally, only four genes used in our lung adenocarcinoma model are currently included in other cancer gene panels, and only one of them is included in all three panels (Supplementary Table 6). This suggests that the majority of genes used in our model is not well-recognized as cancer-associated genes. Since the mutational profile of these 24 lung adenocarcinoma model genes was shown to be highly associated with the responses to cancer immunotherapy and mutation load, the role of these genes in cancer development and progression should be elucidated in future studies.

The genes used in our lung adenocarcinoma mutation load estimation model have a total CDS length of 187,188, which is much shorter than that in the commercial or institutional gene panels.^11,25,26^ Therefore, this represents an additional advantage of a gene panel developed based on our mutation load estimation model, since panel cost depends on the total lengths of the genes selected. Our model should help decrease the cost and time required for panel analysis, which will further accelerate the establishment of diagnosis and medical decisions. Additionally, since there are many gene transcripts, and the CDS length information retrieved from the Ensembl BioMart represents the length of the transcripts, the CDS length of the longest transcript was used when selecting the candidate genes. Therefore, if the panels are developed using the most common transcript of each gene, the total CDS length and cost can be further reduced. Moreover, mutational hotspots can be considered as well when developing a gene panel to minimize the cost.

Although the cost can vary across different platforms, panel designs, analysis pipelines, and practices, we believe a customized targeted gene panel based on our 24-gene lung adenocarcinoma model may be a cost-effective solution for the mutation load estimation and prediction of responses to cancer immunotherapy in lung adenocarcinoma patients. A previous study directly compared the costs of a targeted sequencing panel (Einstein_v1, with a targeted region of 4.98 Mb) to whole-exome sequencing, using the same sequencing platform.^27^ The cost of Einstein_v1 was shown to be approximately one-fourth lower than that of the whole-exome sequencing (USD $281.25 vs. $1266). The targeted region in our 24-gene model (approximately 0.2 Mb) is much smaller than that in Einstein_v1, and the cost can be anticipated to reduce further. Additionally, targeted gene panel approach shortens the turnaround time. A previous study estimated that the data processing CPU time for a 90-gene panel is one-twentieth of that needed for the whole-exome sequencing (5 vs. 100 h).^28^ Furthermore, targeted gene panel approach can substantially increase the throughput, because of its high multiplexing capabilities. For example, in the aforementioned study comparing Einstein_v1 and whole-exome sequencing, whole-exome sequencing allowed only three samples per lane, whereas the targeted sequencing panel Einstein_v1 can analyze 16 samples per lane.^27^ These are all important issues determining clinical applicability of a test.

When we applied the mutation estimation model trained using lung adenocarcinoma data on the melanoma patients, its performance was not as good as that observed for the lung adenocarcinoma patients. Since there are considerable differences in the mutational landscapes between different types of cancer, this was not surprising, and cancer-type-specific mutation load estimation model was shown to be necessary to estimate precisely the mutation load in different types of cancer. We demonstrated that the *R*^2^ between the estimated mutation load and actual mutation load in melanoma patients is higher when using the melanoma model compared with that obtained when using the lung adenocarcinoma model. Additionally, we showed that the colorectal cancer model can precisely estimate the mutation load in colorectal cancer patients, where mutation load estimation is currently not available. However, we noted that the prediction accuracy of the treatment response in melanoma patients with the melanoma model is not superior to that of the lung adenocarcinoma model. This may be due to the relatively weaker association between the mutation load and treatment response in melanoma patients compared with that in the lung adenocarcinoma patients, and mutation load alone may not be sufficient to predict clinical benefits in the melanoma patients, which agrees with the previously obtained results.^17,20^ To elucidate this issue further, we plotted the ROC for clinical benefit prediction using the actual mutation loads of patients treated with anti-CTLA-4/anti-PD-1 immunotherapy (Supplementary Fig. 11), and the AUCs for this classification were shown to be 0.6587 and 0.6092, respectively. Furthermore, the accuracy of clinical benefit prediction using actual mutation load in melanoma patients was shown to be 0.6149 and 0.6842 for anti-CTLA-4 and anti-PD-1 treatments, respectively. These moderate performance indices indicate that the predictive value of mutation load for the treatment response in melanoma patients is not as high as that in the lung adenocarcinoma patients. Consequently, although the melanoma model can be used for the estimation of the mutation load as precisely as the lung adenocarcinoma model, their accuracy in predicting the treatment response is not comparable. This suggests that the ability to predict treatment response for a mutation estimation model depends upon its precision in estimating the mutation load and the nature of the disease as well. The development of different approaches may be necessary to predict immunotherapy treatment response in different types of cancer in future.

The limitation of our study is a relatively small number of cases, since the immunotherapy treatment response data for lung adenocarcinoma patients included only 28 cases,^10^ and therefore, a larger number of cases is required for the validation of the performance of the treatment response prediction. Furthermore, the datasets in this study were mostly obtained in the Caucasian population, and the performance of our model in other ethnicities should be tested. Recently, in addition to the mutation load, other features such as microsatellite instability and neoantigen burden emerged as potential predictive biomarkers for cancer immunotherapy treatment response as well.^29–31^ Therefore, the strategies that integrate different features may be more effective biomarkers for the accurate prediction of cancer immunotherapy response in future.^32^

In summary, we have proposed a computational framework and successfully constructed a mathematical model using only 24 genes that can be used to estimate the mutation load in lung adenocarcinoma precisely. The estimated mutation load can be used to predict the clinical outcome of cancer immunotherapy with high accuracy. Therefore, a customized panel for the targeted sequencing of these selected genes can be designed, instead of whole-exome sequencing. Consequently, by using our mutation load estimation model, the cost and time needed for the assessment of the mutation load should considerably decrease and the cancer immunotherapy response prediction should be more obtainable in the standard clinical setting.

## Materials and methods

### Data used for model construction

Genomics data, specifically somatic mutation information, were used for the construction of the mutation load estimation model. As the training data for the construction of the lung adenocarcinoma model, the somatic mutation data were downloaded from TCGA database (*n* *=* 230).^15^ As the validation data, the somatic mutation data from two independent studies were retrieved (*n* *=* 181 for Imielinski et al.;^16^ *n* *=* 30 for Rizvi et al.,^10^ excluding four patients with squamous cell carcinoma). Additionally, we retrieved the data showing the treatment responses to anti-PD-1 immunotherapy.^10^ For the melanoma model, the somatic mutation data was obtained from TCGA database (*n* = 333)^21^ as the training data. The somatic mutation information from four independent studies (*n* = 333)^17–20^ and clinical outcomes of melanoma patients treated with anti-CTLA-4 (Snyder et al. (*n* = 64)^17^ and Van Allen et al. (*n* = 110)^18^) or anti-PD-1 therapy (Hugo et al. (*n* = 38)^20^) were used as the validation data for the melanoma model. For the colorectal model, as the training data, the somatic mutation data obtained from TCGA database (*n* = 536)^22^ were used, while the validation data were the mutation data retrieved from two independent studies (*n* *=* 619 for Giannakis et al.^23^; *n* *=* 72 for Seshagiri et al.^24^).

### Selection of nonsynonymous point mutations and the construction of mutation matrix

Since the number of nonsynonymous point mutations has been demonstrated to be associated with the clinical benefits of immunotherapy,^10^ the first step was selecting nonsynonymous point mutations from the training data downloaded from TCGA. Here, the column “Variant_Classification” indicates the translational effect of a variant. There are 11 different types of variant classification in TCGA lung adenocarcinoma somatic mutation data and three of them, including nonsense mutation, nonstop mutation, and missense mutation, are considered nonsynonymous point mutations. The mutations of these three types were selected and used for mutation matrix construction. Mutation matrix is an *m* × *n* matrix where *m* indicates the number of genes and *n* represents the number of patients. Each element in the mutation matrix specifies the number of nonsynonymous point mutations in a gene in one patient. Following the selection of the nonsynonymous point mutations, the “Variant_Type” information in TCGA somatic mutation raw data, showing variant types, was used for the calculation of mutation count. The types of variants used here were single-nucleotide polymorphism (SNP), double-nucleotide polymorphism (DNP), and tri-nucleotide polymorphism (TNP), indicating the mutations in one, two, or three consecutive nucleotides, respectively. Therefore, the mutation count calculation was one, two, and three for SNP, DNP, and TNP, respectively. The summation of all mutation counts of a gene in a patient represented the total number of nonsynonymous point mutations. For example, three SNPs, two DNPs, and one TNP in a gene A of a patient gave ten nonsynonymous point mutations in gene A. In this way, the number of nonsynonymous point mutations in each gene for each patient was calculated, generating the mutation matrix.

### Candidate gene selection

There are about 20,000 genes in human genome,^33^ and it is impractical to consider all genes with nonsynonymous point mutations for the model construction. Therefore, candidate genes, which may help estimate the mutation load precisely were selected based on the following three characteristics: mutation frequency, CDS length, and the association between mutation status and mutation load (Fig. 1). For each gene in the mutation matrix, the mutation frequency, i.e., the percentage of patients with mutation in one gene, can be calculated. If the constructed model comprises genes with low mutation frequency, more genes are required for the precise estimation of the mutation load, and, to avoid this, we selected the genes with mutation frequency higher than or equal to 10%. Since we aimed to reduce the cost of mutation load estimation, and the cost of the customized panel is proportional to the number of selected genes and their corresponding CDS lengths, genes with the large CDS lengths were avoided when constructing the model. Here, the CDS lengths for each gene were obtained from the Ensembl BioMart database,^34^ and genes with the CDS lengths larger than 15,000 nucleotides were excluded from further analysis. Furthermore, we aimed to select the mutation load-associated genes that can be used to precisely estimate the mutation load of the patients, and for those where the mutation load was shown to be significantly different between the patients with mutations in a particular gene and the patients with the wild-type gene, these genes were identified as the mutation load-associated gene and selected as potential candidate genes. For example, based on the mutation information of the gene A in the mutation matrix, the patients can be separated into two groups: the mutated group, in which the patients carry the mutation in gene A, and the wild-type group, where the patients do not carry gene A mutations. Wilcoxon rank sum test was employed to test the difference in the mutation loads between these two groups. The genes with Bonferroni corrected *p*-values lower than 0.05 were identified as the mutation load-associated genes and selected as potential candidate genes. The genes that met all three criteria were selected as the candidate genes for further model construction.

### Construction of the mutation load estimation model

Based on the selected candidate genes, a linear mathematical model was used to estimate the mutation load:

2 $$y_m{\mathrm{ = }}c{\mathrm{ + }}\mathop {\sum}\nolimits_{i = 1}^n {a_i \cdot x_{mi}} {\mathrm{ + }}e_m$$

where *y*_*m*_ is the mutation load of the *m*-th patient, *x*_*mi*_, *i* = 1, …, *n*, indicates the mutation count of the selected model gene *i* in the *m*-th patient, *a*_*i*_, *i* = 1, …, *n*, represents the weighting of each selected model gene *i* on the mutation load, *c* specifies the constant term, and *e*_*m*_ is the model uncertainty for the *m*-th patient. The equation shows that the mutation load of a patient can be calculated using the mutation counts of the selected model genes multiplied by the corresponding weightings and adding the constant term and the model uncertainty.

In the mutation load estimation model shown in equation (2), the mutation load *y*_*m*_ and the mutation counts of the selected genes *x*_*mi*_ can be obtained from the generated mutation matrix. On the other hands, the weighting of each selected gene *a*_*i*_ and the constant term *c* represent the model parameters that had to be identified. Subsequently, least squares parameter estimation method was employed for parameter identification and BIC was used for model selection. BIC is a model selection criterion widely used in the field of system identification.^35^ It measures the trade-off between the estimated error and model complexity. The model with the lower value of BIC can estimate the mutation load more precisely without including too many genes in the model. Therefore, the model with the minimal BIC statistics was selected as the most appropriate mutation load estimation model. Details are presented in Supplementary Methods.

### Performance evaluation and validation

We have selected the most appropriate model containing *p* genes with the minimal BIC value. Afterward, we evaluated the performance of the mutation load estimation model by calculating *R*^2^ between the estimated and actual mutation load using the independent validation data. Furthermore, based on the PFS/OS information and the estimated mutation load for each patient, a survival analysis comparing patients with high/low estimated mutation loads was used to determine if the estimated mutation load correlates with the clinical outcome of immunotherapy. Since the immunotherapy response data for lung adenocarcinoma were obtained from Rizvi et al.,^10^ the estimated mutation loads were employed to discriminate between the patients with DCB or NDB as well. The ROC curve was plotted to determine the optimal discrimination threshold and the AUC was calculated. Subsequently, the patients with the estimated mutation load higher than the optimal discrimination threshold were predicted to have DCBs. In contrast, the patients with the estimated mutation load below the optimal discrimination threshold were predicted to have NDBs. In this way, the sensitivity, specificity, and accuracy of the classification were evaluated. Furthermore, we compared the performance of our model with the performances of random models composed of *p* randomly selected genes. Therefore, 10,000 random models with *p* genes were constructed and their performance were evaluated. We generated the empirical distributions of *R*^2^ between the estimated and actual mutation load, AUC statistic of classifier, accuracy of classification for 10,000 random models, and the empirical *p-*values showing the performance of our mutation load estimation model were determined.

### Statistical analysis

Differences in mutation loads were examined by using the Mann–Whitney *U*-test or the Kruskal–Wallis exact test. The log-rank test was used to compare Kaplan–Meier survival curves. Cox proportional-hazards regression model was used to estimate hazard ratios and their associated 95% confidence intervals.

### Data availability

All data used in this study were publicly available prior to analysis (Materials and methods).

### Code availability

The code for mutation load estimation model construction is available upon request.

## Electronic supplementary material

Supplementary Information
