# Supplementary material for: Mutation load estimation model as a predictor of the response to cancer immunotherapy
Source: NPJ Genom Med. 2018 Apr 30;3:12. doi: 10.1038/s41525-018-0051-x (PMC5928060; doi:10.1038/s41525-018-0051-x)
Supplement: Supplementary file 1 — Supplementary Information [file 41525_2018_51_MOESM1_ESM.pdf]

Supplementary Information

Mutation load estimation model as a predictor of the response to cancer immunotherapy

Guan-Yi Lyu[1], Yu-Hsuan Yeh[2], Yi-Chen Yeh[3,4]*, and Yu-Chao Wang[1,5]*

[1]Institute of Biomedical Informatics, National Yang-Ming University, Taipei 11221, Taiwan; [2]Department of Life Sciences and Institute of Genome Sciences, National Yang-Ming University, Taipei 11221, Taiwan; [3]Department of Pathology and Laboratory Medicine, Taipei Veterans General Hospital, Taipei 11217, Taiwan; [4]School of Medicine, National Yang-Ming University, Taipei 11221, Taiwan; [5]Center for Systems and Synthetic Biology, National Yang-Ming University, Taipei 11221, Taiwan

*Correspondence: Yu-Chao Wang (yuchao@ym.edu.tw) or Yi-Chen Yeh (lordaaa@gmail.com)

Supplementary Methods
Supplementary References
Supplementary Figures 1-11
Supplementary Tables 1-6

Supplementary Methods

Construction of the mutation load estimation model

Based on the mutation load estimation model in equation (2) in the main text, least squares parameter estimation method was employed for parameter identification and Bayesian information criterion (BIC) was used for model selection. Considering the mutation information available for m patients, the mutation load estimation model can be expressed in the matrix form as follows:

$$\begin{bmatrix} y_1 \\ y_2 \\ \vdots \\ y_m \end{bmatrix} = \begin{bmatrix} 1 & x_{11} & \cdots & x_{1n} \\ 1 & x_{21} & \cdots & x_{2n} \\ \vdots & \vdots & \ddots & \vdots \\ 1 & x_{m1} & \cdots & x_{mn} \end{bmatrix} \begin{bmatrix} c \\ a_1 \\ \vdots \\ a_n \end{bmatrix} + \begin{bmatrix} e_1 \\ e_2 \\ \vdots \\ e_m \end{bmatrix}. \quad \text{(S1)}$$

For simplicity, the notations Y, X, θ, and E represent the matrices in equation (S1) and the model can be represented by the linear regression model as follows:

$$Y = X\theta + E, \quad \text{(S2)}$$

where $Y = \begin{bmatrix} y_1 \\ y_2 \\ \vdots \\ y_m \end{bmatrix}$, $X = \begin{bmatrix} 1 & x_{11} & \cdots & x_{1n} \\ 1 & x_{21} & \cdots & x_{2n} \\ \vdots & \vdots & \ddots & \vdots \\ 1 & x_{m1} & \cdots & x_{mn} \end{bmatrix}$, $\theta = \begin{bmatrix} c \\ a_1 \\ \vdots \\ a_n \end{bmatrix}$, and $E = \begin{bmatrix} e_1 \\ e_2 \\ \vdots \\ e_m \end{bmatrix}$, respectively. Afterward, the least squares parameter estimation method, which minimizes the sum of squared error (SSE), was applied to identify the parameters[1]:

$$\hat{\theta} = \left(X^T X\right)^{-1} X^T Y, \quad \text{(S3)}$$

where $\hat{\theta}$ is the identified parameter vector of the model.

Assuming we selected k candidate genes, this gives $2^k - 1$ combinations of gene sets, each of which can be constructed as a mutation load estimation model, and the selection of the most appropriate model is necessary. Since the addition of a parameter to the model always decrease the SSE, using the model with more parameters allows a more precise estimation of the mutation load. However, the model with an increased number of parameters may result in overfitting. Additionally, since we aimed to use a small set of genes that can precisely estimate the mutation load, both model complexity and model accuracy must be considered when selecting the most appropriate model. Bayesian information criterion (BIC), which includes both the estimated error and model complexity in one statistics, was employed for model selection[2,3]. BIC formula can be expressed as the following equation:

$$\text{BIC} = m \cdot \ln\left(\frac{\text{SSE}}{m}\right) + n \cdot \ln(m), \quad \text{(S4)}$$

where m is the number of data points (patients) and n is the number of parameters, i.e., the number of selected genes and the constant term. The SSE in equation (S4) can be calculated as

$$\mathrm{SSE} = \sum_{i=1}^{m} \left(y_i - \hat{y}_i \right)^2 , \tag{S5}$$

where y_i is the actual mutation load of the i-th patient, and $\hat{y}_i$ indicates the estimated mutation load of the i-th patient.

Equation (S4) shows that BIC can be separated into two parts: the first term represents the estimated error by SSE, while and the second term considers the model complexity by presenting the number of parameters. In this way, BIC decreases as SSE decreases and increases as the number of parameters increases. As SSE decreases with the increasing parameter numbers for non-adequate model complexities, there should be a minimum around the most appropriate parameter number. Therefore, BIC can be used for the model selection based on the selected genes and their corresponding parameters identified in each model. Due to the computational efficiency, it is impractical to compute the BIC statistics for all $2^k - 1$ possible models. Stepwise methods such as forward selection method and backward elimination method were developed to avoid the complexity of exhaustive search[4,5]. Here, the stepwise regression method, combining the backward elimination method with forward selection method, was applied for the model selection with BIC statistics. Using this, we selected the mathematical model with the minimal BIC value as the most appropriate mutation load estimation model.

Supplementary References

1 Johansson, R. *System modeling and identification*. (Prentice Hall, 1993).

2 Schwarz, G. Estimating the dimension of a model. *Ann. Statist.* **6**, 461-464 (1978).

3 Wit, E., Heuvel, E. v. d. & Romeijn, J.-W. ‘All models are wrong...’: an introduction to model uncertainty. *Statistica Neerlandica* **66**, 217-236 (2012).

4 Hocking, R. R. The analysis and selection of variables in linear regression. *Biometrics* **32**, 1-49 (1976).

5 Draper, N. R. & Smith, H. *Applied regression analysis*. 3rd edn, (Wiley, 1998).

Supplementary Figures

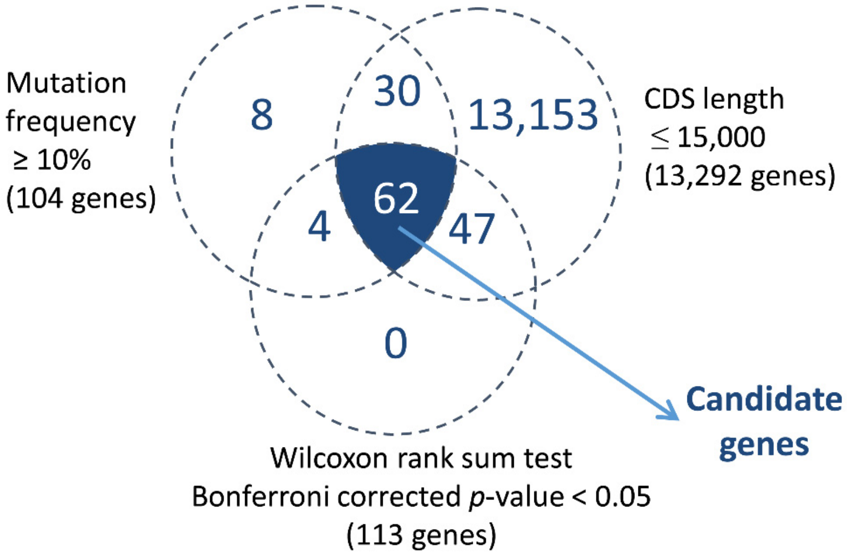

Supplementary Figure 1. Venn diagram of three criteria for candidate gene selection. The number of genes and overlaps which meet the corresponding criteria are shown in the diagram. Sixty-two genes which meet all three criteria were selected as the candidate genes.

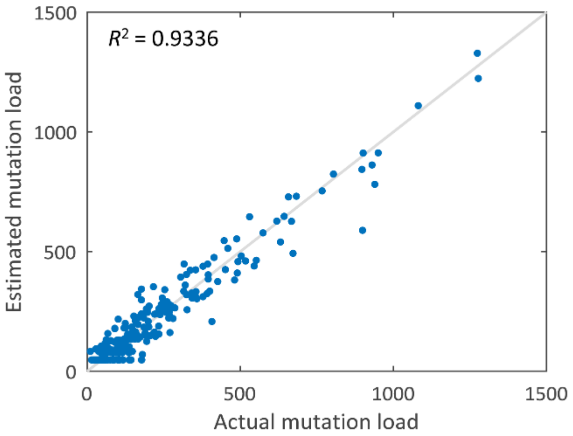

Supplementary Figure 2. Estimated mutation load versus actual mutation load using the training data of lung adenocarcinoma from TCGA (n = 230). R^2 = 0.9336.

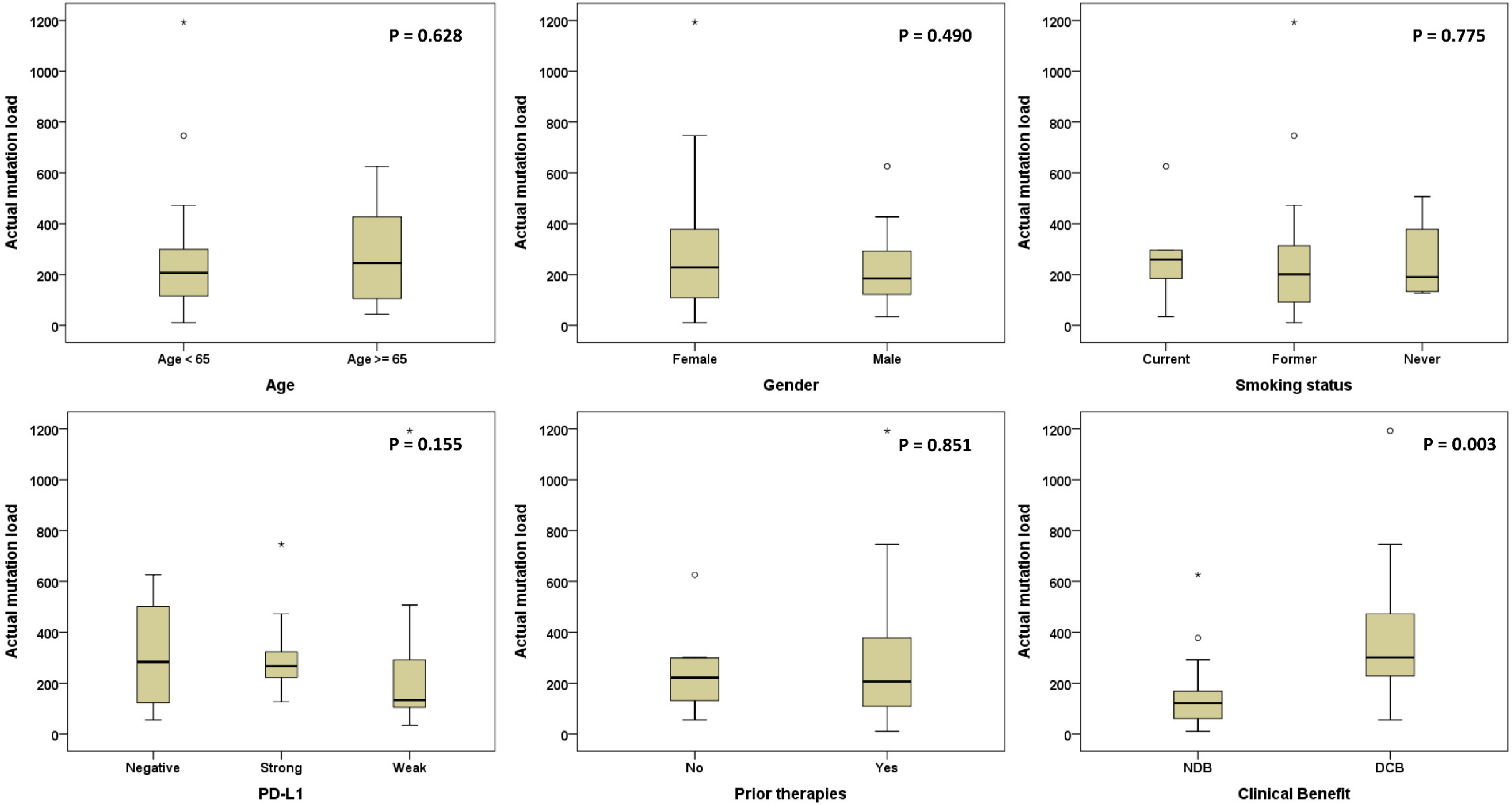

Supplementary Figure 3. Actual mutation load of patient subgroups with different clinical characteristics in the lung adenocarcinoma patient cohort.

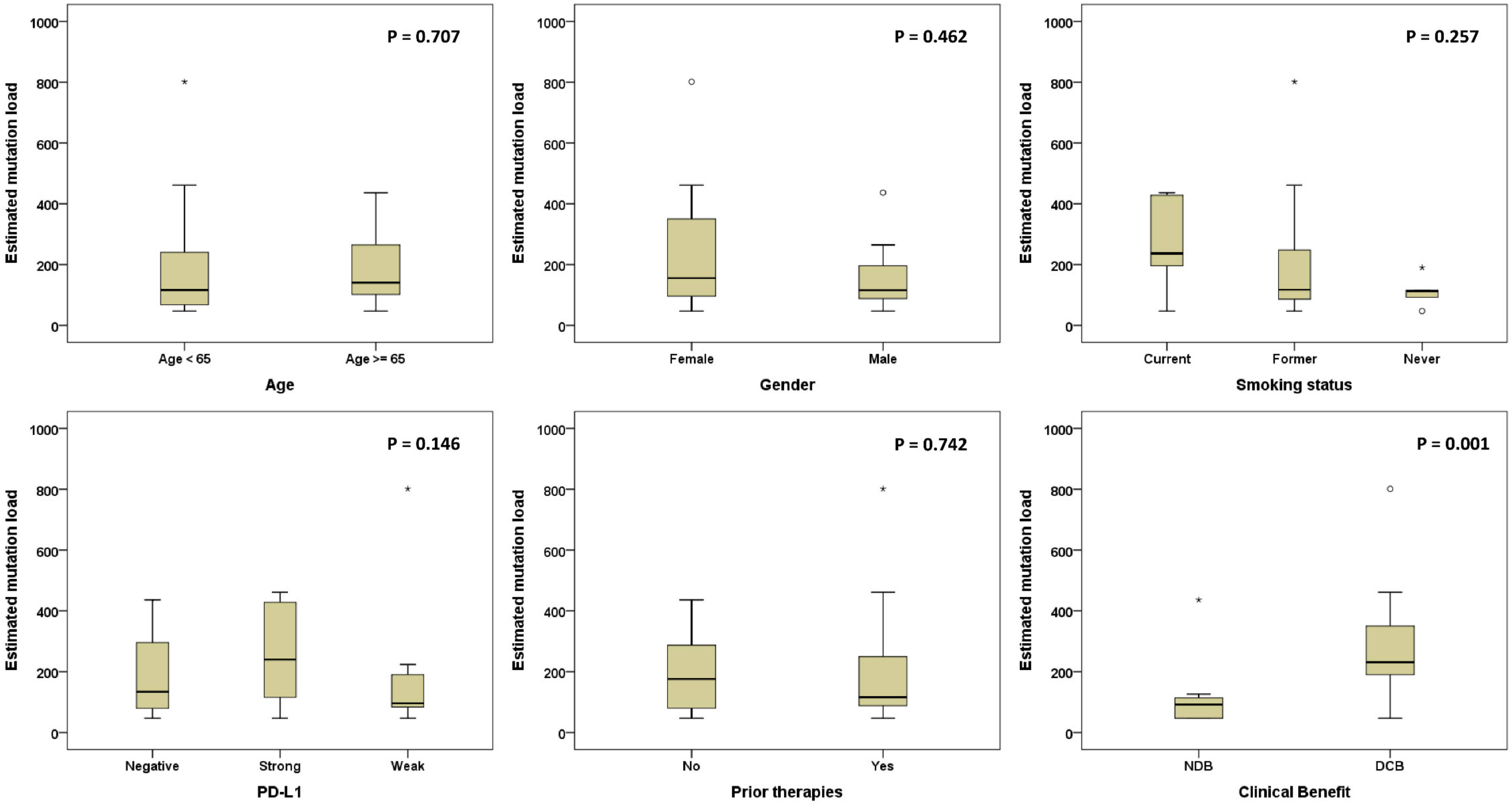

Supplementary Figure 4. Estimated mutation load of patient subgroups with different clinical characteristics in the lung adenocarcinoma patient cohort.

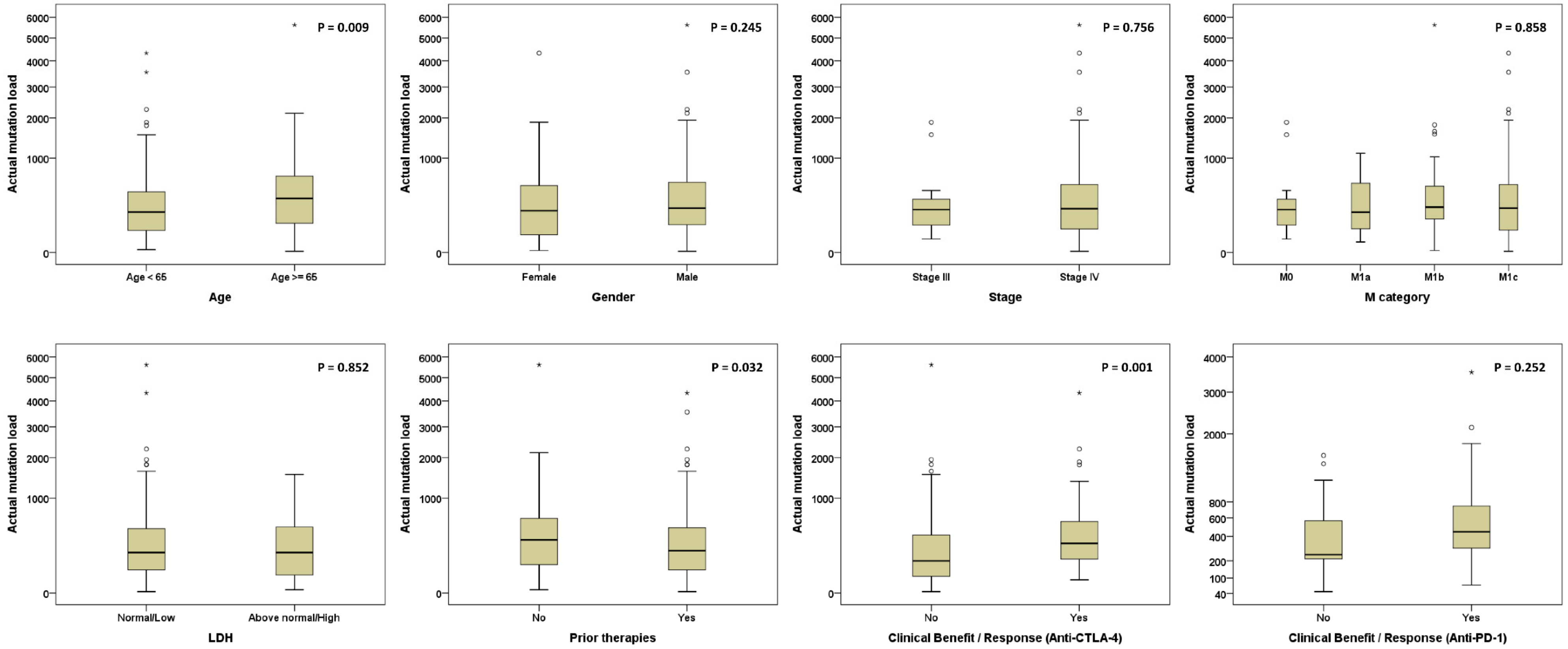

Supplementary Figure 5. Actual mutation load of patient subgroups with different clinical characteristics in the melanoma patient cohort.

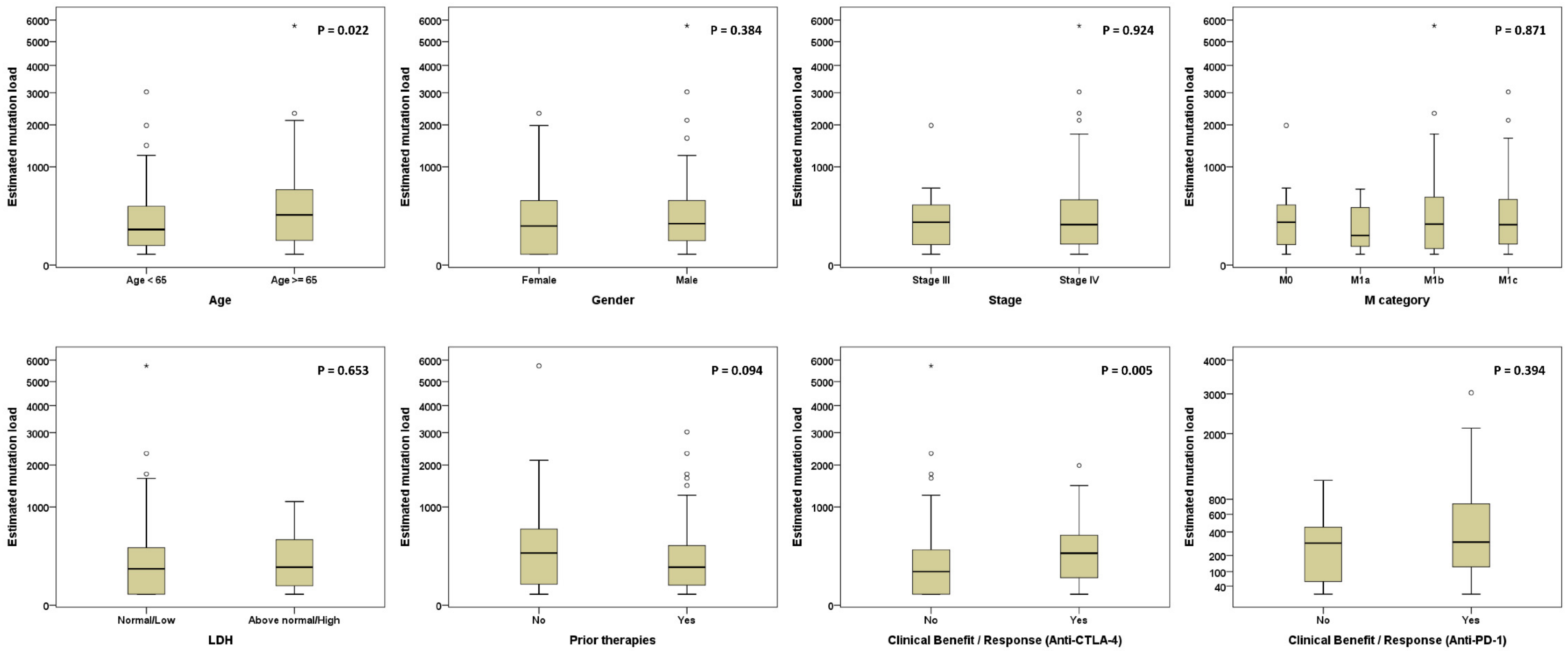

Supplementary Figure 6. Estimated mutation load of patient subgroups with different clinical characteristics in the melanoma patient cohort.

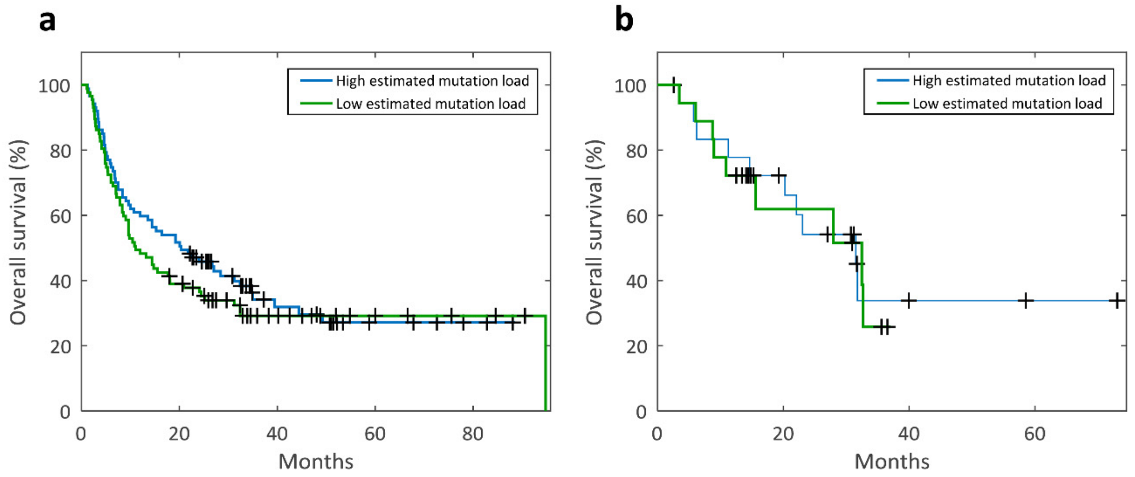

Supplementary Figure 7. Survival analysis for melanoma patients treated with anti-CTLA-4/anti-PD-1 immunotherapy. (a) Survival analysis comparing OS in anti-CTLA-4 treated melanoma patients with the high estimated mutation loads (n = 87) and those with the low estimated mutation loads (n = 87). The estimated mutation load is not significantly associated with OS (p = 0.3473, log-rank test). (b) Survival analysis comparing OS in anti-PD-1 treated melanoma patients with the high estimated mutation loads (n = 19) and those with the low estimated mutation loads (n = 18). The estimated mutation load is not significantly associated with OS (p = 0.8873, log-rank test).

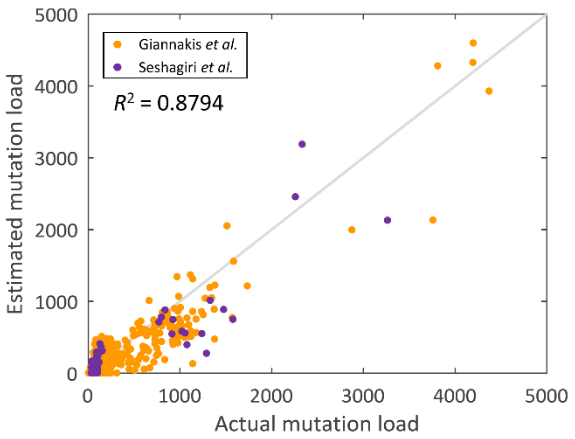

Supplementary Figure 8. Estimated mutation load versus actual mutation load using the colorectal model in an independent validation cohort (n = 691). R^2 = 0.8794.

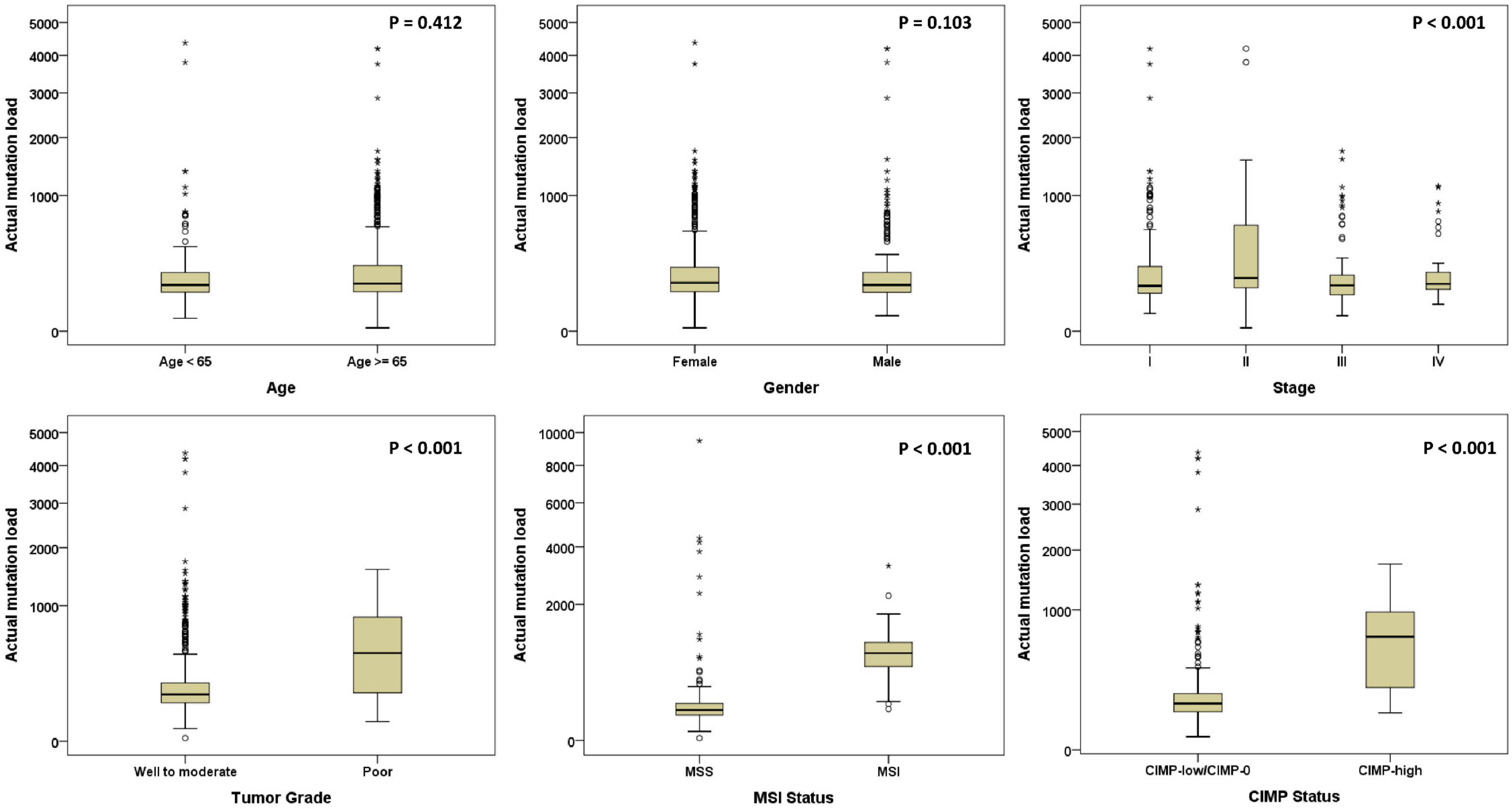

Supplementary Figure 9. Actual mutation load of patient subgroups with different clinical characteristics in the colorectal cancer patient cohort.

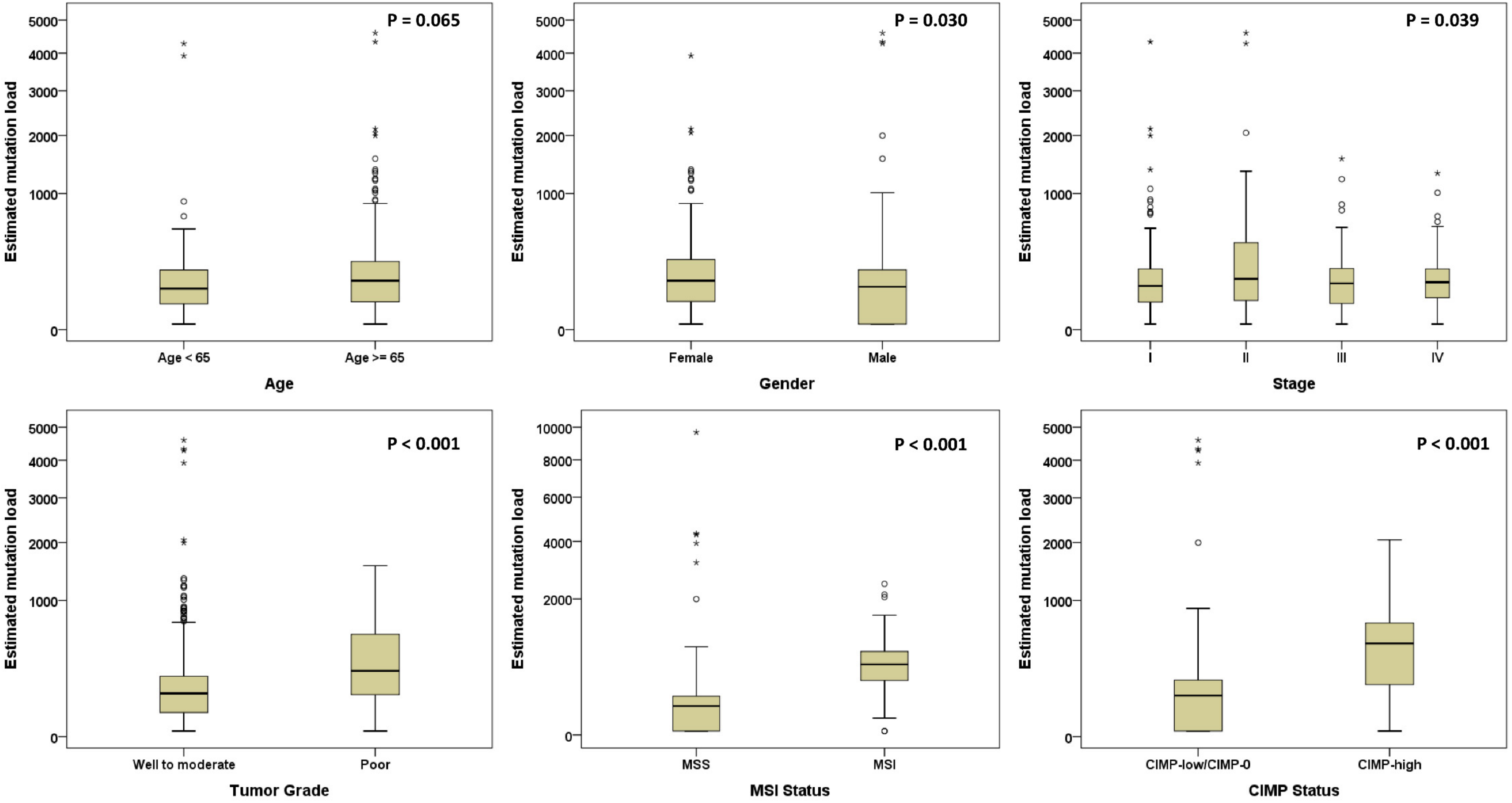

Supplementary Figure 10. Estimated mutation load of patient subgroups with different clinical characteristics in the colorectal cancer patient cohort.

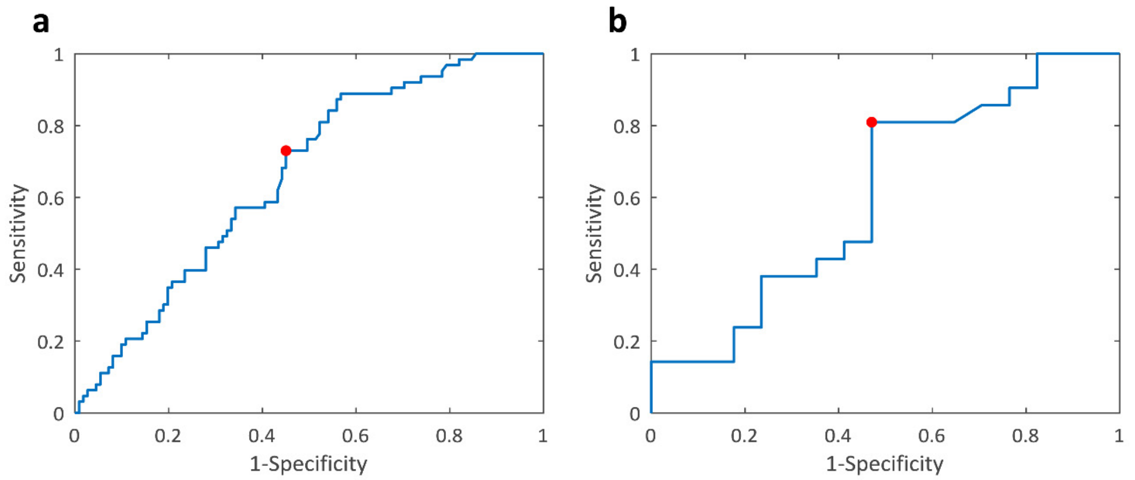

Supplementary Figure 11. ROC curves for the classification of clinical benefit using the actual mutation load in melanoma patient treated with anti-CTLA-4/anti-PD-1 immunotherapy. (a) ROC curve for anti-CTLA-4 treatment (n = 174). Red point, the optimal discrimination threshold 180. AUC = 0.6587. (b) ROC curve for anti-PD-1 treatment (n = 37). Red point, the optimal discrimination threshold 265. AUC = 0.6092.

Supplementary Tables

Supplementary Table 1. Selected candidate genes and related information for lung adenocarcinoma model

Gene symbol	Entrez ID	Mutation frequency	CDS length*	Wilcoxon *p*-value
ADAMTS12	81792	16.09%	4785	3.87805E-10
ADGRG4	139378	16.52%	9243	2.87403E-10
ADGRL3	23284	13.04%	4743	4.92669E-08
ANK2	287	14.78%	11874	3.35196E-07
APOB	338	16.52%	13692	2.14214E-10
ASPM	259266	13.04%	10434	2.26852E-07
ASTN1	460	13.04%	3885	8.09295E-07
ASXL3	80816	15.22%	6747	4.31808E-07
BRINP3	339479	12.17%	2301	1.02715E-07
CACNA1E	777	13.91%	6942	2.73033E-08
CDH10	1008	14.78%	2367	7.51573E-07
CDH12	1010	10.43%	2385	6.66325E-07
CNTNAP2	26047	11.74%	3996	4.59839E-08
COL11A1	1301	15.65%	5457	8.21948E-10
COL6A3	1293	12.17%	9534	1.12296E-06
CPS1	1373	10.87%	4521	1.7754E-07
CSMD2	114784	10.87%	10896	7.39118E-09
CSMD3	114788	34.35%	11124	7.25254E-13
DNAH3	55567	11.30%	12351	1.8322E-06
DNAH9	1770	13.48%	13461	3.61178E-09
ERICH3	127254	12.17%	4593	2.67253E-06
FAM135B	51059	12.61%	4221	2.36022E-06
FAM47C	442444	10.43%	3108	2.97689E-07
FAT4	79633	14.78%	14946	1.00835E-08
FBN2	2201	12.61%	8739	7.12594E-10
FLG	2312	26.96%	12186	3.43821E-13
FLG2	388698	16.09%	7176	7.44336E-10
HRNR	388697	13.48%	8553	3.83618E-07
KMT2C	58508	18.26%	14736	2.35005E-08
LRP1B	53353	28.26%	13800	8.60476E-16
LRP2	4036	10.87%	13968	9.68643E-10
MUC17	140453	20.00%	13482	1.52909E-09

MXRA5	25878	14.78%	8487	1.0128E-10
NALCN	259232	14.35%	5217	1.43453E-07
NAV3	89795	18.70%	7158	9.15102E-08
NOTCH4	4855	10.00%	6018	1.34932E-07
NRXN1	9378	12.61%	4644	1.87759E-08
PAPPA2	60676	15.65%	5376	7.73413E-07
PCDH11X	27328	11.30%	4044	1.53451E-07
PCDH15	65217	17.83%	5895	2.42856E-11
PKHD1L1	93035	10.00%	12732	4.14314E-07
PLPPR4	9890	10.00%	2292	4.14314E-07
PRDM9	56979	12.17%	2685	1.19391E-06
PTPRD	5789	13.91%	5739	1.04936E-08
PXDNL	137902	13.04%	4392	2.69664E-07
RELN	5649	15.22%	10383	2.92322E-09
RIMS2	9699	10.43%	4104	1.79872E-06
RP1L1	94137	16.52%	7203	3.83611E-08
RYR2	6262	33.04%	14904	2.67988E-12
RYR3	6263	10.43%	14622	5.29954E-08
SI	6476	15.65%	5484	6.17042E-08
SORCS1	114815	10.87%	3507	3.14108E-07
SPATA31A6	389730	11.30%	4032	5.34076E-07
SPATA31D1	389763	10.87%	4731	7.82685E-08
TAF1L	138474	13.04%	5481	3.06299E-08
TNR	7143	13.48%	4077	3.43262E-06
TPTE	7179	10.00%	1656	1.13666E-07
XIRP2	129446	19.13%	10650	7.2114E-10
ZFHX4	79776	26.52%	10851	6.76814E-12
ZNF536	9745	17.39%	3903	5.3449E-08
ZNF804A	91752	15.65%	3630	3.83686E-09
ZNF831	128611	11.30%	5034	3.05446E-07

* CDS length information is retrieved from Ensembl BioMart database

Supplementary Table 2. Univariate and multivariate Cox regression analysis for progression-free survival in lung adenocarcinoma patients treated with anti-PD-1 therapy

Variable	Univariate analysis			Multivariate analysis (with actual mutation load)			Multivariate analysis (with estimated mutation load)		
	HR	95% CI	P-Value	HR	95% CI	P-Value	HR	95% CI	P-Value
**Age**									
**< 65**	Ref								
**≧65**	1.037	0.412-2.608	0.939						
**Gender**									
**Male**	Ref								
**Female**	0.456	0.188-1.107	0.083						
**Smoking status**									
**Never**	Ref								
**Former**	0.352	0.119-1.039	0.059						
**Current**	0.303	0.071-1.291	0.106						
**PD-L1 expression**									
**Negative/Weak**	Ref			Ref			Ref		
**Strong**	**0.139**	0.032-0.612	**0.009**	**0.203**	0.043-0.958	**0.044**	**0.087**	0.016-0.468	**0.004**
**Prior courses of chemotherapy**									
**No**	Ref								
**Yes**	0.804	0.481-1.345	0.407						
**Actual mutation load**									

**Low**	Ref			Ref					
**High**	**0.202**	0.071-0.575	**0.003**	0.380	0.123-1.170	0.092			
**Estimated mutation load**									
**Low**	Ref						Ref		
**High**	**0.188**	0.069-0.513	**0.001**				**0.126**	0.033-0.486	**0.003**

Supplementary Table 3. Univariate and multivariate logistic regression analysis for clinical benefit / treatment response in melanoma patients treated with anti-CTLA-4 therapy

**Variable**	**Univariate analysis**			**Multivariate analysis (with actual mutation load)**			**Multivariate analysis (with estimated mutation load)**		
	**OR**	**95% CI**	**P-Value**	**OR**	**95% CI**	**P-Value**	**OR**	**95% CI**	**P-Value**
**Age**									
**< 65**	Ref								
**≧65**	1.675	0.898-3.125	0.10.5						
**Gender**									
**Male**	Ref								
**Female**	0.830	0.426-1.614	0.582						
**Stage**									
**Stage III**	Ref								
**Stage IV**	0.324	0.101-1.038	0.058						
**M category**									
**M0**	Ref			Ref			Ref		
**M1a**	0.446	0.090-2.215	0.324	0.488	0.044-5.416	0.559	0.358	0.041-3.142	0.354
**M1b**	0.500	0.130-1.930	0.315	0.601	0.105-3.446	0.567	0.754	0.152-3.750	0.730
**M1c**	**0.283**	0.087-0.921	**0.036**	0.397	0.083-1.905	0.248	0.428	0.103-1.779	0.243
**LDH**									
**Low**	Ref			Ref			Ref		
**High**	**0.476**	0.226-1.005	**0.051**	0.490	0.211-1.140	0.098	0.498	0.219-1.134	0.097
**Prior courses of systemic therapy**									

**No**	Ref			Ref			Ref		
**Yes**	**0.487**	0.244-0.973	**0.042**	0.503	0.210-1.203	0.123	0.572	0.252-1.297	0.181
**Actual mutation load**									
**Low**	Ref			Ref					
**High**	**6.095**	2.551-14.561	**< 0.001**	**9.104**	2.919-28.395	**< 0.001**			
**Estimated mutation load**									
**Low**	Ref						Ref		
**High**	**2.955**	1.554-5.616	**0.001**				**2.794**	1.327-5.883	**0.007**

Supplementary Table 4. Characteristics of the melanoma patients responding or non-responding to anti-PD-1 therapy

	Non-responding (n=17)	Responding (n=21)	Total (n=38)	P-value
**Age (mean, yrs)**	56.71±17.28	63.62±11.10	60.53±14.42	0.144#
**Gender**				
**Male**	11 (40.7%)	16 (59.3%)	27 (100%)	0.491*
**Female**	6 (54.5%)	5 (45.5%)	11 (100%)	
**M category**				
**M0**	0 (0%)	1 (100%)	1 (100%)	0.922*
**M1a**	3 (60.0%)	2 (40.0%)	5 (100%)	
**M1b**	2 (50.0%)	2 (50.0%)	4 (100%)	
**M1c**	12 (42.9%)	16 (57.1%)	28 (100%)	
**Prior MAPK-targeted therapy**				
**No**	11 (45.8%)	13 (54.2%)	24 (100%)	0.859*
**Yes**	6 (42.9%)	8 (57.1%)	14 (100%)	
**Median actual mutations (Range)**	244 (45-1556)	447 (69-3549)	441 (45-3549)	0.252&
**Median estimated mutations (Range)**	325 (18-1085)	306 (18-3027)	298 (18-3027)	0.394&

#: Student's t-test

*: chi-squared test or Fisher's exact test

&: Mann-Whitney U test

Supplementary Table 5. Genes and the corresponding parameters used in the constructed colorectal mutation load estimation model.

Gene symbol	Entrez ID	Parameter	Gene symbol	Entrez ID	Parameter
DNAH10	196385	140.26	FAT1	2195	79.07
BRAF	673	139.55	DNAH8	1769	69.82
DNAH3	55567	117.70	FAT4	79633	62.16
FREM2	341640	110.72	UNC80	285175	61.32
DNAH17	8632	108.77	LRP1B	53353	52.12
FAT2	2196	105.28	CSMD1	64478	51.50
DNAH5	1767	99.88	DNAH11	8701	50.11
DYNC2H1	79659	86.24	ANK2	287	48.86
COL6A3	1293	85.22	PCDH17	27253	47.94
CACNA1E	777	83.79	DCHS2	54798	46.10
TENM3	55714	79.12	RYR2	6262	36.31
			Constant term		4.02

Supplementary Table 6. The comparison of genes in our constructed lung adenocarcinoma model with other cancer gene panels

Gene symbol	Entrez ID	MD Anderson Cancer Center (170)	Foundation Medicine (315)	Hospital Sírio-Libanês (641)
ADAMTS12	81792			
ADGRG4	139378			
ASTN1	460			
ASXL3	80816			
COL6A3	1293			
CSMD2	114784			
ERICH3	127254			
FAM135B	51059			
FAM47C	442444			
FLG	2312			√
HRNR	388697			
KMT2C	58508		√	√
LRP1B	53353	√	√	√
LRP2	4036			
MXRA5	25878			√
NALCN	259232			
NOTCH4	4855			
NRXN1	9378			
PAPPA2	60676			
PLPPR4	9890			
PXDNL	137902			
RYR3	6263			
ZFHX4	79776			
ZNF831	128611			

The number in parentheses indicates the number of genes included in each panel.
